# Dysregulation of Ketone Body Metabolism Is Associated With Poor Prognosis for Clear Cell Renal Cell Carcinoma Patients

**DOI:** 10.3389/fonc.2019.01422

**Published:** 2019-12-17

**Authors:** Wanmeng Cui, Wenqi Luo, Xiaohui Zhou, Yunliang Lu, Wenqing Xu, Suhua Zhong, Guofei Feng, Yushan Liang, Libin Liang, Yingxi Mo, Xue Xiao, Guangwu Huang, Liudmila Matskova, Zhe Zhang, Ping Li, Xiaoying Zhou

**Affiliations:** ^1^Key Laboratory of High-Incidence-Tumor Prevention & Treatment, Ministry of Education, Guangxi Medical University, Nanning, China; ^2^Department of Pathology, Guangxi Medical University Cancer Hospital, Nanning, China; ^3^Life Science Institute, Guangxi Medical University, Nanning, China; ^4^Institute of Living Systems, Immanuel Kant Baltic Federal University, Kaliningrad, Russia; ^5^Department of Pathology, College & Hospital of Stomatology, Guangxi Medical University, Nanning, China

**Keywords:** clear cell renal cell carcinoma, ketone metabolism, prognosis, bioinformatic analysis, tumor suppressor

## Abstract

Kidney is an important organ for ketone body metabolism. However, the role of abnormal ketone metabolism and its possible function in tumorigenesis of clear cell renal cell carcinoma (ccRCC) have not yet been elucidated. Three differentially expressed key enzymes involved in ketone body metabolism, ACAT1, BDH2, and HMGCL, were screened out between ccRCC and normal kidney tissues using the GEO and TCGA databases.We confirmed that the transcription and protein expression of ACAT1, BDH2, and HMGCL were significantly lower in ccRCC by real-time RT-PCR and IHC assays. Those patients with lower expression of these three genes have a worse outcome. In addition, we demonstrated that ectopic expression of each of these genes inhibited the proliferation of ccRCC cells. The overexpressed *ACAT1* and *BDH2* genes remarkably impeded the migratory and invasive capacity of ccRCC cells. Furthermore, exogenous β-hydroxybutyrate suppressed the growth of ccRCC cells *in vitro* in a dose-dependent manner. Our findings suggest that *ACAT1, BDH2*, and *HMGCL* are potential tumor suppressor genes, and constitute effective prognostic biomarkers for ccRCC. Ketone body metabolism might thus be a promising target in a process for developing novel therapeutic approaches to treat ccRCC.

## Introduction

Abnormal energy metabolism, as a result of reprogrammed metabolic pathways, has been acknowledged as one of the hallmarks of cancer (1). The metabolites produced by altered metabolism serve as an important biochemical driver to maintain the malignant phenotype of cancer. Alteration in glycometabolism, known as the “Warburg effect,” described as the preferential fermentation of glucose to lactate even under sufficient oxygen supply, is common in malignancies. Strategies that target glycolysis have become diagnostic and therapeutic approaches to treat cancer patients (2, 3). Recently, active fatty acid metabolism has been revealed in tumor cells. Accelerated synthesis and β-oxidation of fatty acids provide essential sources of bioenergy, as well as biosynthetic precursors and cell signaling molecules for tumor cells, thereby facilitating cell division and proliferation (4, 5).

Ketone metabolism has gradually become a hot topic of molecular and clinical cancer research (6–8). During fasting, the liver can rapidly oxidize and decompose fatty acids to form acetyl-CoA, further synthesize ketone bodies, thereby providing a fast and efficient energy supply to important energy-consuming organs such as brain, muscle, and kidney. Though ketogenesis is an alternative metabolic pathway in normal cells, ketone bodies and their derivatives like cholesterol and esters have been shown to contribute to tumorigenesis (9). Fatty acids are transported into mitochondria via carnitine palmitoyltransferase (CPT-1) pathway and are broken down into acetyl-CoA via β-oxidation. Two acetyl-CoA molecules are fused to form acetoacetyl-CoA by acetyl-CoA acetyltransferase (ACAT) action and subsequently converted to HMG-CoA by the action of HMG-CoA synthase (HMGCS). Another enzyme-HMG-CoA lyase (HMGCL), converts HMG-CoA to acetoacetate, which is then converted by β-hydroxybutyrate dehydrogenase (BDH) to produce β-hydroxybutyrate (10).

The dysregulation of ketone metabolism in various kinds of tumors remains a controversy. In malignant brain tumors, pancreatic cancer, prostate cancer, breast cancer, colon cancer, stomach cancer, lung cancer, etc., down-regulation of genes involved in ketone metabolism was reported (11). Ketone acetoacetate has been shown to inhibit the growth of colon cancer and breast cancer cells and to reduce the accumulation of intracellular ATP, a process mediated by uncoupling protein 2 (12). It was found that ketone bodies inhibit the growth of human neuroblastoma cells and accelerate their apoptosis (13). Ketone bodies diminished the rate of glycolysis and promote the apoptosis of pancreatic cancer cells, suggesting that ketone body might reduce the malignancy of pancreatic cancer cells (14). In animal experiments, the addition of ketone bodies was shown to inhibit the growth of mouse metastatic tumor cells and prolong the survival of mice with metastatic cancer. The ketone body can inhibit cancer *in vitro* and *in vivo* independently of the blood sugar level and caloric restriction (15). In 2015 (11), it was suggested that ketogenic diet could be the basic treatment of metabolic disorders. The ketogenic diet can be used as an auxiliary treatment method in tumor-bearing mouse models. The diet facilitated a reduction in the size of the tumors and a prolongation of the survival time of the tumor-bearing mice. The authors suggested that the ketogenic diet increased the blood ketone content, strengthened the β-oxidation in the body, lowered the blood sugar level, and then reduced the rate of glycolysis, affecting cell proliferation and inhibiting tumor growth (11). This suggests that changes in ketone metabolism may be one of the factors in tumor pathogenesis. Thus, therapeutic intervention to regulate ketone metabolism may rightly be an effective approach for tumor therapy. However, details of ketone metabolism in tumor cells, and the molecular mechanisms underlying these changes have not been elucidated yet. At the same time it was shown that in liver cancer, transformed hepatoma cells utilize ketones body as energy supply to promote tumor development by changing metabolic characteristics and metabolic modes in the absence of nutrients (16).

Renal cell carcinoma (RCC) is a malignant tumor that occurs in renal tubular epithelial cells. Approximately 208,500 new cases of RCC are diagnosed worldwide each year, accounting for 2% of all cancers (17). The three most common subtypes of RCC are clear cell RCC (ccRCC) (~70%), papillary RCC (10~15%), and chromophobe RCC (~5%) (18, 19). The incidence of kidney cancer in smokers is twice than that of non-smokers (20), suggesting that smoking might be a contributing factor for developing RCC. Other risk factors include genetics, obesity, and high blood pressure, etc (21).

Ketone body metabolism is an essential pathway to convert nutrients in the kidney (22, 23). At present, the role of ketone body metabolism in kidney cancer development remains poorly understood. In this study, we have identified and analyzed the contribution of three differentially expressed genes involved in ketone body metabolism to ccRCC development. Our data suggest that renal cell carcinoma could be classified into subtypes depending on the activity of these three genes, which would open a perspective for a more precise and effective treatment of ccRCC.

## Materials and Methods

### Differentially Expressed Genes (DEGs) Analysis

The cDNA microarray data (GSE36895), which includes 28 cases of ccRCC patients and 21 cases of non-cancer patients, were downloaded from gene expression omnibus (GEO) datasets (https://www.ncbi.nlm.nih.gov/geo). A fold change >5, with cut-off values *p* < 0.05 and *q* < 0.05 was set to screen out DEGs. The heatmap was generated using Cluster software.

### TCGA Data Analysis Using UCSC Xena Browser

The mRNA-Seq expression data from 533 ccRCC patients and 72 non-cancerous kidney tissues were downloaded using UCSC Xena browser (http://xena.ucsc.edu/) to analyze genes expression and their correlation with clinical parameters of ccRCC patients. Based on this dataset, the correlation between the expression of each of two genes, the Kaplan-Meier plot to evaluate overall survival and disease-free survival rates were analyzed.

### Immunohistochemical Analysis

A tissue microarray including 85 primary ccRCC tissues and matched adjacent non-cancerous kidney tissues was purchased from Shanghai Outdo Biotech Co., Ltd. (Shanghai, China; Cat no: HPro-Ade180PG-01).

Paraffin sections (4 mm) of samples were deparaffinized and antigen retrieval was performed in citrate buffer for 3 min at 100°C. The samples were subjected to proteolytic digestion and blockage of an endogenous peroxidase, then slides were incubated overnight with primary antibody against ACAT1 (HPA004428), BDH2 (HPA036028), and HMGCL (HPA004727) from Sigma Aldrich (St. Louis, MO, USA), at a dilution of 1:200, 1:800, 1:600, respectively at 4°C. Then, secondary antibody (ZB-2305, ZSGB-BIO, Beijing) labeled with horseradish peroxidase (HP) was applied for 1 h at room temperature. Slides were soaked in a substrate for antibody-bound HP to initiate the immunochemical reaction. Hematoxylin was used for counterstaining. Images were acquired using a microscope (C-5050, Olympus, Japan). Normal liver tissue was used as a positive control.

Following mounting with neutral gums, immunostaining was scored by 2 independent experienced pathologists, who evaluated slides without knowing the clinicopathological data and clinical outcomes for the patients. The scores of the 2 pathologists were compared and any discrepancies in the scores were resolved by the re-examining of the staining by both pathologists to achieve a consensus score. The immunolabeling of the cancer cells was evaluated. The number of positively stained cells in 5 representative microscopic fields was counted and the percentage of positive cells was also calculated. Cytoplasmic staining was regarded as a positive signal accordingly to the antibody specification sheet. Tumor specimens were scored in a semi-quantitative manner taking into account the heterogeneity of the staining of the target proteins. Protein levels were evaluated by two criteria: the percentage of positive staining (i.e., from 0 to 100%) and intensity level of staining [i.e., “0” (negative), “1” (faint yellow), “2” (yellow or deep yellow), and “3” (tan or brown)] in each tumor sample. A final immunoreactivity score (IRS) was calculated as multiplication of two values: the percentage of positive staining and the intensity level for each tumor sample.

### Quantitative Real-Time PCR Analysis

For quantitative real-time PCR (qRT-PCR) analysis, a cDNA microarray (*n* = 30) containing 14 primary ccRCC tissues cDNA and 14 adjacent non-cancerous kidney tissues cDNAs were obtained from Shanghai Outdo Biotech Co., Ltd. (Shanghai, China; Cat no: me cDNA-HKidE030CS01). The cDNAs were amplified and relative expression levels in different samples were detected by qRT-PCR using the Power SYBR Green PCR Master Mix and a QuantStudio 5 Real-Time PCR System (Foster City, CA, USA, Applied Biosystem). After the reactions were completed, the comparative threshold cycle (Ct) method was used to calculate the relative gene expression. The sequences of primers used were as follows: *ACAT1*-Forward, 5′-GGCTGGTGCAGGAAATAAGA-3′, *ACAT1*-Reverse, 5′-GGAATCCCTGCCTTTTCAAT-3′; *BDH2*-Forward, 5′- GCTTCCAGCGTCAAAGGAGTT-3′, *BDH2*-Reverse, 5′-CAGTTGCGAATCTTCCCGTC-3′; *HMGCL*-Forward, 5′- ACCACCAGCTTTGTGTCTCC-3′; *HMGCL*-Reverse, 5′-GAGGCAGCTCCAAAGATGAC-3′; *GAPDH*-Forward, 5′-AAGCTCACTGGCATGGCCTT-3′; *GAPDH*-Reverse, 5′-CTCTCTTCCTCTTGTGCTCTTG-3′.

### Plasmid, Cell Lines, and Transfection

The full-length coding sequence for *ACAT1, BDH2*, and *HMGCL* were subcloned into the pCMV6-Entry vector obtained from Origene (Rockville, MD, USA).

The ccRCC cell line 786-0 (CRL-1932, ATCC, USA) was maintained in 1640 medium (Invitrogen, Carlsbad, CA, USA) supplemented with 10% fetal bovine serum (FBS; Invitrogen) in an atmosphere with 5% CO_2_ at 37°C.

Cells were transiently transfected with an X-tremeGENE HP DNA transfection reagent (Roche, Mannheim, Germany) accordingly to the manufacturer's instructions. Cells were collected after 48 h of culturing for further experiments.

### Cell Proliferation Assay

Cells (1 × 10^3^ cells per well) were seeded into 96-well plates and allowed to grow for 5 days to build a growth curve. Cell density was examined every 24 h by use of Cell Counting Kit-8 (CCK-8) assay (CK04, Dojindo, Shanghai), in a plate reader at OD450 nm absorbance wavelength (iMark, Bio-Rad, Hercules, CA, USA). Each experiment was performed in quintuplicate.

### Wound Healing Assay

Wound healing assay was performed in ibidi chambers (Culture-Inserts 2 Well, ibidi, Germany) in a 24-well plate. The 6 × 10^5^ cells in the volume of 70 microliters were added to each well. After culturing for 24 h in a cell culture incubator, the culture-inserts were taken out. We can saw the scratches at the location where the culture-inserts were removed. The width of the scratch was evaluated visually at 2-time points (0 and 12 h) by light microscopy (CKX41, Olympus, Japan) and measured with Image J software. The experiment was performed in triplicate.

### Transwell Assay

Cells (1 × 10^5^ per well) resuspended in serum-free 1640 medium were seeded in the upper chamber of 24-well Bio-Coat Invasion Chambers (BD, Bedford, MA, USA) coated with Matrigel. The lower chamber was filled with 1640 medium with 10% FBS. Non-invaded cells on the upper surface of the membranes were removed by using a cotton-tipped swab after 24 h of culture. The migrated, invasive cells on the lower surface of the membrane were fixed with 1% paraformaldehyde for 30 min. Finally, the membrane was stained with 0.1% crystal violet for 30 min and photographed under a microscope. The stained cells photographed were counted using software (Photoshop, Adobe, USA). The migrated and invasive cells on the surface of the inferior membrane were fixed with 4% tissue fixative for 30 min. Finally, it was stained with 0.1% crystal violet for 30 min and photographed under a microscope (CKX53, Olympus, Japan).

### Determination of Human β-Hydroxybutyrate Concentration

Human β-hydroxybutyrate ELISA kit (#JL 19218, JiangLai Biolog, Shanghai, China) was used to measure the concentration of intracellular and extracellular β-hydroxybutyrate. 5 × 10^5^ cells were lysed with 50 μl of cell lysis buffer (Invitrogen, Thermo Fisher Scientific, Austria). The procedure was followed strictly by instructions. Finally, samples were read in a plate reader at OD450 nm absorbance wavelength (iMark, Bio-Rad, Hercules, CA, USA). Three replicates were set for each sample.

### Statistical Analysis

Statistical analyses were performed by using IBM SPSS Statistics 22 software for Windows (SPSS Inc., Chicago, IL, USA). The independent Student's *t*-test was used to analyze the results and data are expressed as the means ± SD. The Mann-Whitney test was used for non-normally distributed data. Statistical analyses were performed using Fisher's exact test for any 2 × 2 tables, the Pearson χ2 test for non-2 × 2 tables, Kaplan-Meier plots for survival analysis and the Cox proportional hazards regression model for univariate and multivariate survival analyses. Differences were considered statistically significant when the *p*-value was <0.05.

## Results

### Expression of *ACAT1, BDH2*, and *HMGCL* Genes Is Down-Regulated in ccRCC

**Figure 1:**
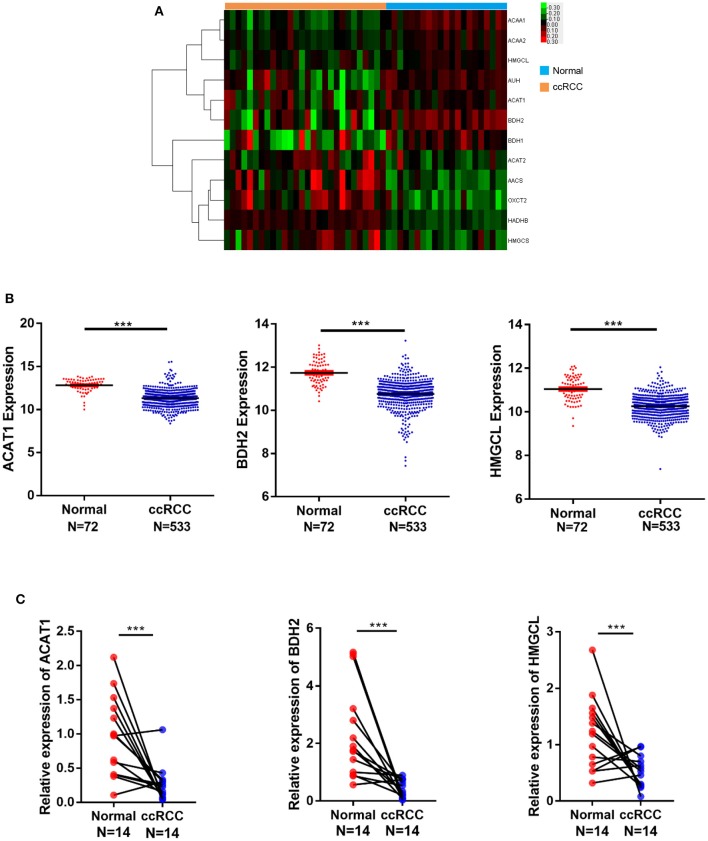
mRNA expression of *ACAT1, BDH2*, and *HMGCL* is downregulated in ccRCC in contrast to normal kidney samples**. (A)** A heatmap of the hierarchical clustering of 12 differentially expressed key genes involved in ketone body metabolism. Input data are the log2 ratios of mRNA expression values for ccRCCs over normal kidney tissues. Genes that are upregulated designated with red color, green color indicate downregulated genes. **(B)** The mRNA expression analysis of *ACAT1, BDH2*, and *HMGCL* genes in 533 cases of ccRCC and 72 cases of normal kidney tissues based on TCGA database. **(C)** The mRNA expression analysis of *ACAT1, BDH2*, and *HMGCL* genes by RT-qPCR in 14 primary renal cell carcinoma tissues and in the matched adjacent tissues, normalized to mRNA GAPDH a house-keeping gene expression level. ***p < 0.001.

To reveal differentially expressed genes participating in fatty acid metabolism in ccRCC, we analyzed cDNA microarray data containing totally 28 cases of ccRCC and 21 cases of normal kidney tissues, for genes selected from GEO database (GSE36895), using the GCBI platform. We found that *ACAT1, BDH2, HMGCL*, the three key genes involved in the ketogenesis and ketolysis, were significantly downregulated in ccRCC (Figure 1A), suggesting an alteration of ketone body metabolism in ccRCC. To confirm this, we analyzed the transcriptional levels of *ACAT1, BDH2*, and *HMGCL* using RNA-seq data downloaded from TCGA, which contained 533 ccRCC cases and 72 normal controls. As shown in Figure 1B, *ACAT1, BDH2*, and *HMGCL* mRNA levels were significantly lower in ccRCC tissues as compared to the matched normal tissues.

To verify the expression of *ACAT1, BDH2*, and *HMGCL* genes on a larger scale, 20 GEO datasets containing 707 ccRCC samples and 387 uncancerous controls were gathered and applied in the succedent meta-analysis. *ACAT1, BDH2*, and *HMGCL* expression patterns in microarray data were summarized in Table S1. Random-effect models were utilized in all three analyses owing to high heterogeneity (*ACAT1*: *p* < 0.01, *I*^2^ = 85.2%; *BDH2*: *p* < 0.01, *I*^2^ = 87.6%; *HMGCL*: *p* < 0.01, *I*^2^ = 93.2%; Figures S1A–C). In accordance to our expectation, the integral transcriptional expression level of *ACAT1, BDH2*, and *HMGCL* all prominently descended in ccRCC in compare with normal groups (*ACAT1*: SMD = −1.89, 95% CI = −2.35 to −1.43; *BDH2*: SMD = −1.78, 95% CI = −2.27 to −1.28; *HMGCL*: SMD = −1.60, 95% CI = −2.25 to −0.95). Besides, sensitivity analysis (Figures S1D–F) noted the absence of a remarkable deviation in each individual study given by the similarity between the original result and those plotted after one GEO chips were omitted. Furthermore, the results of Egger's test for *ACAT1, BDH2*, and *HMGCL* genes were *p* = 0.948> 0.05, *p* = 0.939> 0.05, *p* = 0.992> 0.05, respectively, which indicated no overt publication bias (Figures S1G–I).

To corroborate our findings further, we performed a transcriptional analysis of these genes by qRT-PCR in 14 cases of ccRCC and in the matched control samples. In agreement with our bioinformatical analysis, the *ACAT1, BDH2*, and *HMGCL* mRNA levels were significantly lower in ccRCC in the qRT-PCR assay as well (Figure 1C).

**Figure 2:**
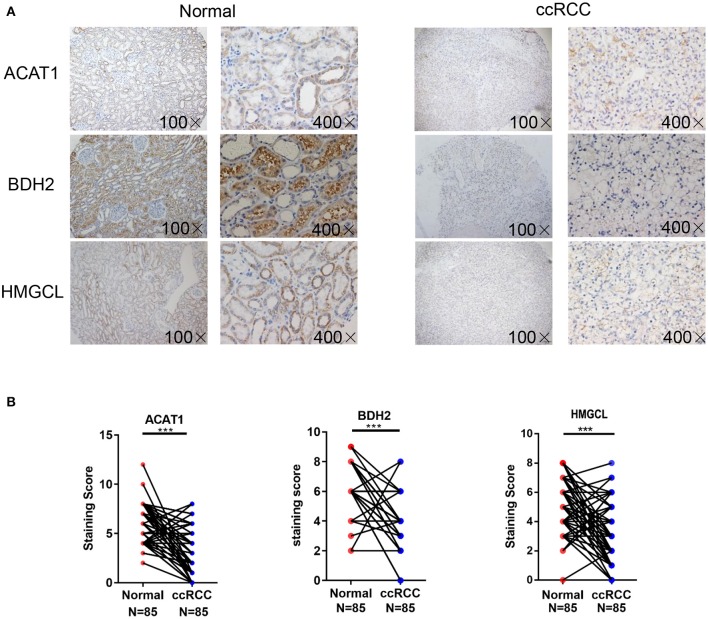
Detection of ACAT1, BDH2, and HMGCL protein expression in 85 cases of primary ccRCC tissues and matched adjacent kidney tissues. **(A)** Immunohistochemical staining. Representative slides of ccRCC and matched adjacent tissues samples were stained by ACAT1, BDH2, and HMGCL antibodies, **(B)** Analysis of IHC staining. Scores of IHC staining of all the investigated ccRCCs and corresponding adjacent non-cancerous stromal tissue samples. ***p < 0.001.

Furthermore, tissue microarrays containing 85 pairs of ccRCC tissue and matched normal kidney samples counterparts were examined for the ACAT1, BDH2, and HMGCL protein expression levels by immunohistochemical (IHC) assay (Figure 2). IHC staining and subsequent quantitative analyses of IHC results revealed that the expression of ACAT1, BDH2, and HMGCL in ccRCC tissues was mainly located in the cytoplasm but was significantly lower than in the corresponding normal kidney tissues. This further supports our findings that expression of ACAT1, BDH2, and HMGCL is dysregulated in ccRCC.

### The *ACAT1, BDH2*, and *HMGCL* mRNAs Expression Levels Are Significantly Intercorrelated

**Figure 3:**
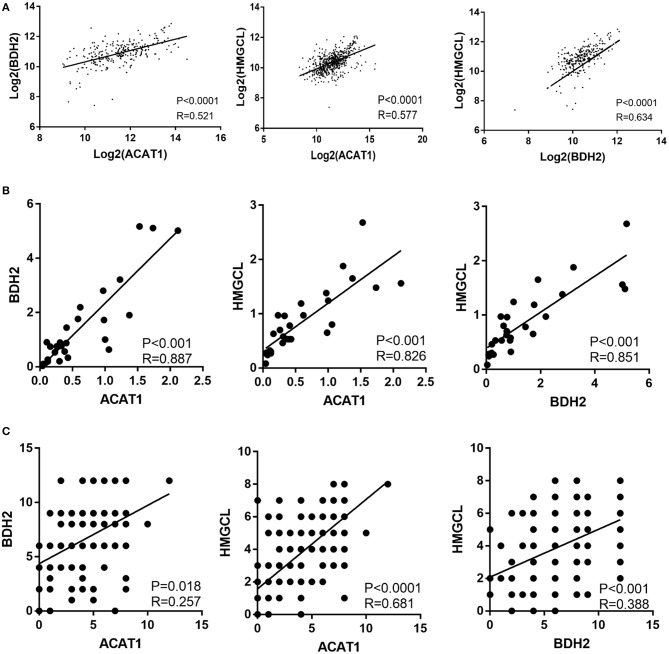
The expression of *ACAT1, BDH2*, and *HMGCL* is significantly inter-correlated in ccRCCs. The pair-wise inter-correlation of transcriptional levels of *ACAT1* and *BDH2, ACAT1* and *HMGCL, BDH2* and *HMGCL* based on TCGA database **(A)** and RT-qPCR data **(B)**. **(C)** The pair-wise inter-correlation of protein levels of ACAT1 and BDH2, ACAT1 and HMGCL, BDH2 and HMGCL based on IHC staining scores.

We investigated the correlation between the mRNA expression levels for the *ACAT1, BDH2*, and *HMGCL* genes in ccRCC based on TCGA database. Interestingly, the expression of each of the three genes was remarkably correlated both in ccRCC and normal control samples (Figure 3A).

We analyzed the pair-wise correlation between the mRNA and protein levels, detected in qPCR and IHC assays respectively, for each of the two of these three molecules. We found a significant pair-wise correlation between the expression levels of these three molecules (Figures 3B,C).

### The Transcriptional Level of *ACAT1, BDH2*, and *HMGCL* Genes Has a Predictive Power as Diagnostic Biomarkers for ccRCC

**Figure 4:**
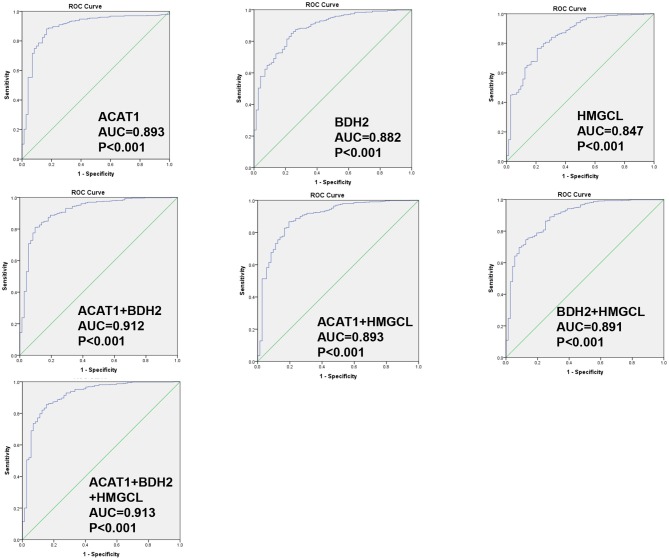
ROC curve for *ACAT1, BDH2*, and *HMGCL* genes in ccRCC patients based on TCGA RNA-seq data. The ROC curves to evaluate the diagnostic value of *ACAT1, BDH2*, and *HMGCL* expression, either independently in 533 cases of RCC and 72 cases of normal kidney tissues, or combining two genes or three genes.

We wondered whether the differential expression levels of *ACAT1, BDH2*, and *HMGCL* genes alone or in a combination could distinguish ccRCC and normal samples. We performed a receiver operating characteristic curve (ROC) analysis of TCGA RNA-seq data. As shown in Figure 4, the area under the curve (AUC) values for *ACAT1, BDH2*, and *HMGCL* mRNAs were 0.893 (95%CI: 0.851–0.935), 0.882 (95%CI: 0.842–0.921), and 0.847 (95%CI: 0.797–0.897), respectively (Table S2). In a combination of *ACAT1* and *BDH2*, the AUC value was 0.912 (95%CI: 0.874–0.951). The AUC value was 0.893 (95%CI: 0.850–0.937) when combining *ACAT1* and *HMGCL*, and was 0.891 (95%CI: 0.850–0.932) when combining *BDH2* and *HMGCL*. Furthermore, analysis of the AUC values for all of the three genes showed the following numbers: 0.913 (95%CI: 0.874–0.952). Our analysis suggests that mRNA levels of *ACAT1, BDH2*, and *HMGCL* could be used as diagnostic markers for ccRCC either alone or in a combination. An analysis of three gene combinations is better for predicting ccRCC.

### The Levels of *ACAT1, BDH2*, and *HMGCL* Expression Significantly Correlates With Clinicopathological Characteristics in ccRCC

We further analyzed the relation of clinicopathological variables and mRNA levels for the three genes based on TCGA RNA-seq data for ccRCC (Table 1). We found that female patients have a higher mRNA level of ACAT1 than males. The lower expression of *ACAT1* and *BDH2* was significantly correlated with stage III-IV, grade 3-4, T3-4 stages, lymph node, and distant organ metastasis, respectively. While *HMGCL* was significantly correlated only with stage III-IV and T3-4 stages. Our data indicate that the expression of ketone body metabolic genes was successively reduced during ccRCC progression.

**Table 1:** Association of *ACAT1, BDH2*, and *HMGCL* expression with clinicopathological characteristics accordingly to TCGA mRNA-seq data.

**Characteristic**	**Cases**	**ACAT1**		**BDH2**		**HMGCL**	
		**Mean ± SD**	***p*-value**	**Mean ± SD**	***p*-value**	**Mean ± SD**	***p*-value**
**Age (years)**							
<60	245	11.291 ± 1.041	0.591	10.733 ± 0.647	0.384	10.278 ± 0.490	0.470
≥60	288	11.338 ± 0.987		10.784 ± 0.696		10.245 ± 0.549	
**Sex**							
Male	345	11.178 ± 1.001	0.000^***^	10.723 ± 0.666	0.083	10.229 ± 0.529	0.065
Female	188	11.567 ± 0.975		10.829 ± 0.686		10.317 ± 0.504	
**Stage**							
I–II	324	11.510 ± 1.004	0.000^***^	10.885 ± 0.562	0.000^***^	10.321 ± 0.506	0.001^**^
III–IV	208	11.016 ± 0.953		10.567 ± 0.784		10.166 ± 0.535	
**Grade**							
G1-G2	243	11.430 ± 0.883	0.003^**^	10.880 ± 0.492	0.000^***^	10.295 ± 0.494	0.126
G3-G4	282	11.179 ± 1.053		10.653 ± 0.785		10.226 ± 0.537	
**T stage**							
T1-T2	342	11.484 ± 1.011	0.000^***^	10.882 ± 0.565	0.000^***^	10.317 ± 0.511	0.001^**^
T3-T4	191	11.015 ± 0.943		10.543 ± 0.791		10.159 ± 0.528	
**Lymph node status**							
N0	240	11.365 ± 1.051	0.006^**^	10.787 ± 0.631	0.000^***^	10.238 ± 0.550	0.409
N1	16	10.625 ± 0.937		9.983 ± 0.775		10.119 ± 0.629	
**Metastasis**							
M0	422	11.386 ± 1.007	0.000^***^	10.778 ± 0.623	0.003^**^	10.266 ± 0.523	0.102
M1	79	10.946 ± 1.031		10.478 ± 0.828		10.161 ± 0.487	

*The gene-level transcription estimates as a log2(x+1) transformed RSEM normalized count*.

** *p < 0.01*;

*** *p < 0.001*.

Furthermore, the relationships between ACAT1, BDH2, and HMGCL protein levels and other clinicopathological parameters was investigated based on the analysis of the IHC staining results (Table S3). The protein expression of BDH2 and HMGCL demonstrated a correlation with TNM stage. In addition, BDH2 protein expression levels are correlated with tumor size. Data from this analysis further supports our findings concerning the mRNA levels for these three proteins in ccRCC. However, we were unable to investigate the correlation of the expression of these molecules with the lymph node status and metastasis due to a lack of information.

### Decreased mRNA Expression of *ACAT1, BDH2*, and *HMGCL* Is Indicative of the Worse Prognosis for ccRCC Patients

**Figure 5:**
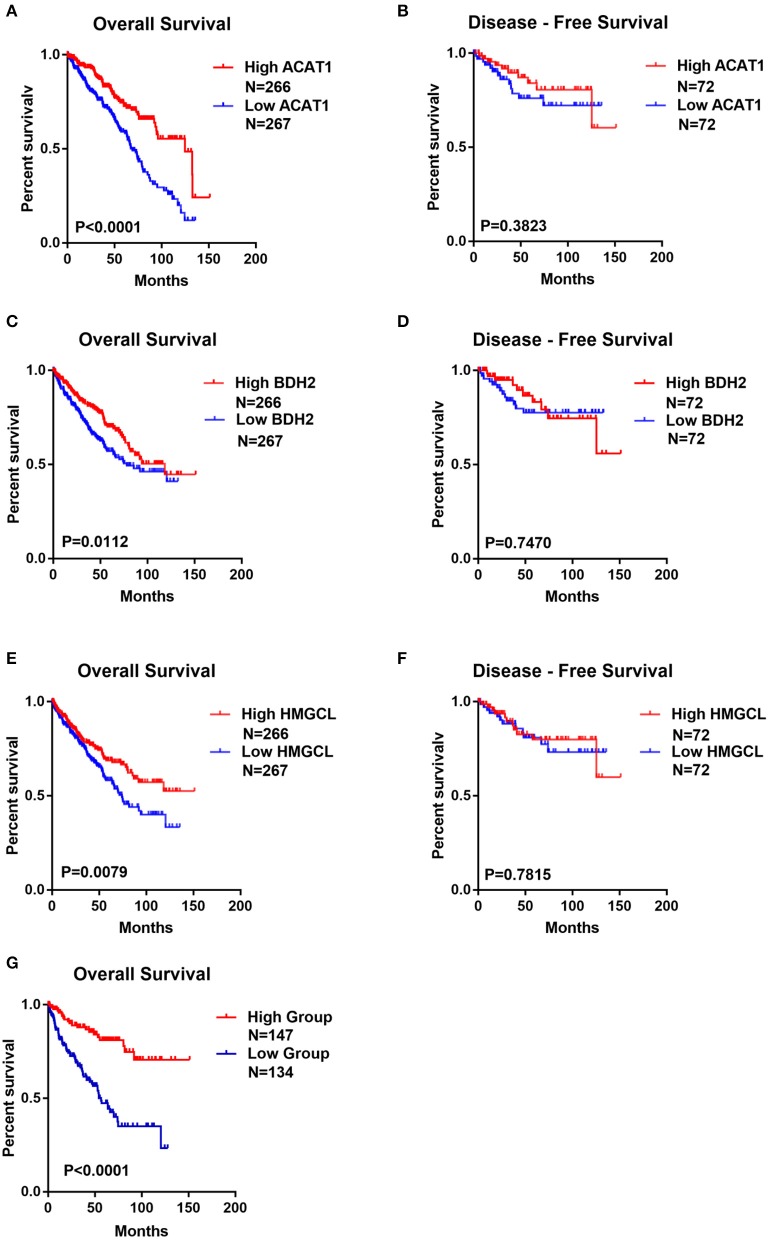
The lower expression of *ACAT1, BDH2*, and *HMGCL* predicts the worse prognosis for ccRCC patients. Kaplan-Meier survival curves of overall survival **(A,C,E)** and disease-free survival **(B,D,F)** were plotted based on the mRNA level of *ACAT1*, BDH2, and *HMGCL*, respectively. **(G)** Kaplan-Meier survival curves for ccRCC patients with combined values of *ACAT1*, BDH2, and *HMGCL* expression based on TCGA mRNA-seq data.

The potentially prognostic significance of *ACAT1, BDH2*, and *HMGCL* expression levels for ccRCC patients was investigated. Kaplan–Meier analysis of the GEPIA database showed that the lower expression of *ACAT1, BDH2*, and *HMGCL* mRNAs correlated both with the worse overall survival (OS, log-rank test, *p* = 4.9e-08, *p* = 2.8e-06, *p* = 2.1e-04) and shorter disease-free survival (DFS, log-rank test, *p* = 9.3e-06, *p* = 8.4e-06, *p* = 7.7e-04) in ccRCC patients, respectively (Figures 5A–F).

We divided ccRCC patients into two groups: one with all the three genes having high expression, which was considered as having active metabolism of ketone bodies. The other group, with the lower expression of these genes, was considered to be impaired in ketone body metabolism. Interestingly, we observed that ccRCC patients with less dynamic metabolism of ketone bodies had remarkably lower OS time (Figure 5G). This data indicates that the status of the ketone body metabolism, characterized by the expression level of *ACAT1, BDH2*, and *HMGCL* genes may be a crucial factor for the outcome of the disease in ccRCC patients.

### Functional Assessment of *ACAT1, BDH2*, and *HMGCL* Genes Activity

**Figure 6:**
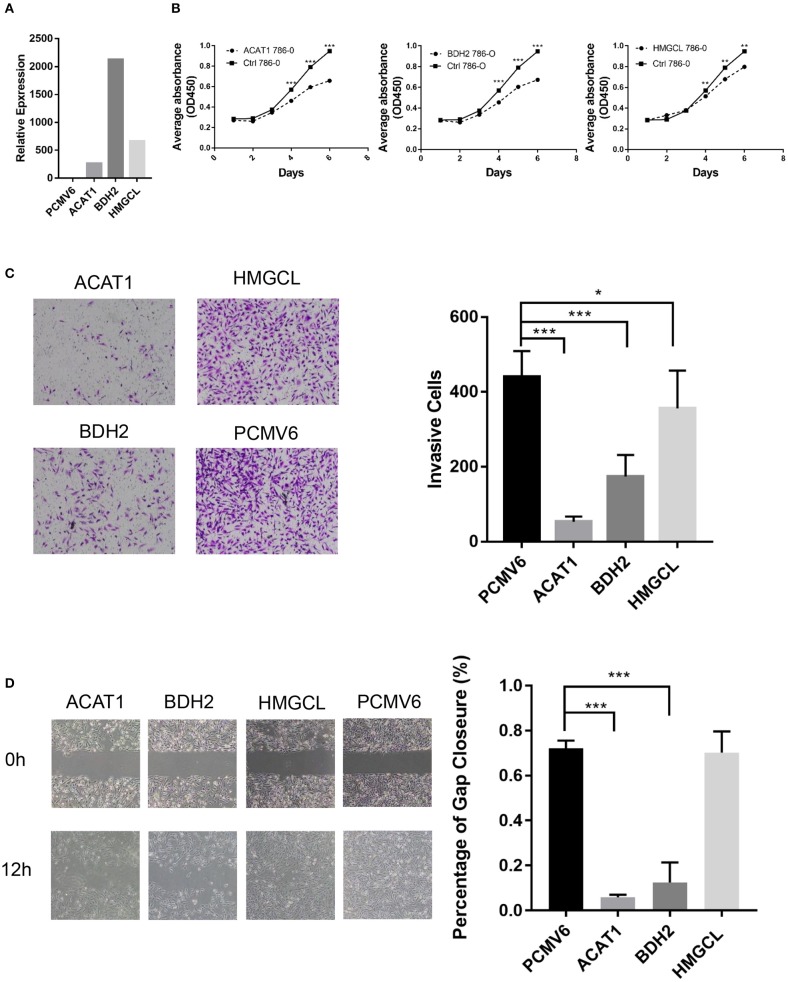
The tumor-suppressive effects of *ACAT1, BDH2*, and *HMGCL* expression in ccRCC. **(A)** The mRNA levels of *ACAT1, BDH2*, and *HMGCL* were determined by a real-time fluorescent quantitative PCR assay after transient transfection of 24 h. A bar chart shows a ratio of an mRNA expression value in cells transfected with a gene-coding vector to an mRNA value in cells transfected with an empty vector pCMV6. **(B)** The proliferation of 786-0 cells restored the expression of *ACAT1, BDH2*, and *HMGCL* genes was measured by a CCK8 assay. **(C)** A transwell assay. The violet color dots represent cells penetrating through matrix gels. **(D)** Wound healing assay. Images were taken at 0 and 12 h after introducing a scratch in 786-0 cells. Gap closure was measured as mean ± SD of three independent experiments. *p < 0.05; **p < 0.01; ***p < 0.001.

To access the function of *ACAT1, BDH2*, and *HMGCL* genes in ccRCC, we ectopically expressed these three genes in the ccRCC cell line 786-0. The expression of *ACAT1, BDH2*, and *HMGCL* genes was confirmed at mRNA level by a real-time RT-PCR assay (Figure 6A). We observed that the *ACAT1*/*BDH2*/*HMGCL*-786-0 transiently transfected cells grew more slowly than the control cells (Figure 6B). In addition, a transwell assay and a wound-healing assay were performed to investigate the effect of *ACAT1, BDH2*, and *HMGCL* expression on invasive and migratory capacity of ccRCC cells. We found that *ACAT1* and *BDH2* overexpressing 786-0 cells invaded the extracellular matrix gel to a much lower extent than the control cells (Figure 6C) and that the gap closure in the transfected cells was slower (Figure 6D), indicating a role of the restored *ACAT1* and *BDH2* expression in attenuation of the metastatic processes in ccRCC disease.

However, restoring the expression of *HMGCL* did not affect the motility of 786-0 cells. These data are in line with our bioinformatics analysis, showing that decreased expression of *ACAT1* and *BDH2* genes but not *HMGCL* significantly correlated with lymph node and distant organ metastasis. Our cell culture data thus support the conclusion that restored expression of *ACAT1, BDH2*, and *HMGCL* genes, suppressed the growth of ccRCC cells, and that *ACAT1, BDH2* but not *HMGCL* impede the metastatic capacity of ccRCC cells. These genes might thus be considered as tumor suppressors during ccRCC progression.

**Figure 7:**
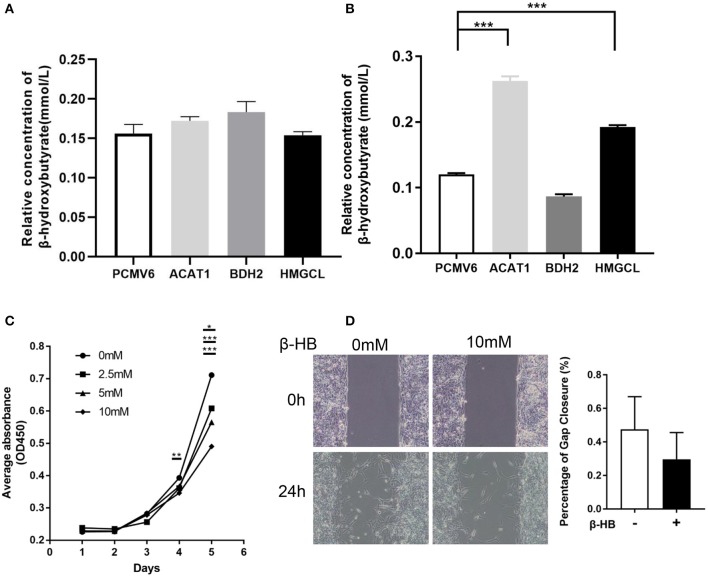
The effect of β-hydroxybutyrate on the proliferation and migration of 768-0 cells. The relative concentration of extracellular **(A)** and intracellular **(B)** β-hydroxybutyrate, when restoring the expression of ACAT1, BDH2, and HMGCL, respectively. **(C)** The proliferation of 786-0 cells was measured by a CCK8 assay upon the treatment of β-hydroxybutyrate with concentration of 0, 2.5, 5, and 10 mM. **(D)** Wound healing assay. Images were taken at 0 and 24 h after introducing a scratch in 786-0 cells. Gap closure was measured as mean ± SD of three independent experiments. *p < 0.05; **p < 0.01; ***p < 0.001.

As the key enzymes regulating ketone body metabolism, we further investigated the role of these three genes in ketone body production. The intracellular and extracellular concentration of β-hydroxybutyrate after restoring the expression of ACAT1, BDH2, and HMGCL, respectively was measured. We found that the relative concentration of β-hydroxybutyrate in the cultured medium was slightly elevated when overexpressing ACAT1 and BDH2, respectively (Figure 7A). Meanwhile, the intracellular β-hydroxybutyrate significantly increased when ACAT1 and HMGCL overexpressed (Figure 7B). We further addressed the effect of β-hydroxybutyrate on ccRCC cells. The proliferation of 768-0 cells was significantly suppressed by β-hydroxybutyrate treatment, in a dose-dependent manner (Figure 7C), and the migrative capacity of 768-0 cells was slightly inhibited by β-hydroxybutyrate (Figure 7D). This suggested that restoring the expression of ACAT1, BDH2, and HMGCL increases the production of β-hydroxybutyrate and leads to growth suppression in 768-0 cells.

## Discussion

There is a great need to uncover the pathogenesis of ccRCC at the molecular level. To date, early inactivation of the *VHL* gene, encoding a ubiquitin ligase, which marks hypoxia-induced factor α (HIF-1α) for proteasomal degradation, thus responsible for cellular oxygen sensing, is pointed out as a key tumor suppressor gene impaired in ccRCC (24–26). The HIF-1α promotes the expression of many genes encoding glycolytic enzymes. The HIF-1α promotes the conversion of pyruvate to lactic acid and, at the same time, blocks pyruvate conversion to acetyl coenzyme A and negatively affects the expression of enzymes involved in oxidative phosphorylation. Thus, HIF-1α counteracts metabolism through the tricarboxylic acid (TCA) cycle (27). In 2016, renal cell carcinoma (RCC) with succinate dehydrogenase (SDH)-deficiency was included in the WHO classification (19). The biallelic mutation of SDH results in inactivation of the SDH enzyme, which is a mitochondrial complex of type II, associated with the respiratory chain and the Krebs cycle, and is now considered to have tumor suppressor properties (28, 29). These are indications that impaired metabolism is one of the causes of the RCC pathogenesis, and analysis of changes in expression of key metabolism-regulating molecules can be used as the basis for the molecular typing of RCC.

In the present study, we found that the mRNA and protein levels of ACAT1, BDH2, and HMGCL are significantly downregulated in ccRCC. The transcriptional inactivation of these genes significantly correlated with the advanced pathological stages and shorter survival time for ccRCC patients. One can speculate that the downregulation of expression of genes responsible for ketone body metabolism results in a reduced amount of ketone bodies.

We observed that an analysis of *ACAT1, BDH2*, and *HMGCL* mRNAs expression in a pairwise combination predicts the outcome for ccRCC patients with better prediction power. We speculate that ccRCC could be classified into subtypes depending on the expression analysis of *ACAT1, BDH2*, and *HMGCL* genes. It is worth to further explore the regulation of activity of these genes, as a new therapeutic approach. Perhaps a ketogenic diet will benefit ccRCC patients with altered ketone metabolism.

β-hydroxybutyrate is the most abundant circulating ketone body representative in the human body. Recently it was reported the role of β-hydroxybutyrate as an endogenous histone deacetylase inhibitor (30). But whether the ketone body metabolism plays a role in the pathogenesis of RCC remains unknown. There are abundant epigenetic alterations in RCC, due to abnormal histone modification, DNA methylation and deregulated microRNAs expression, all of which have been considered as important factors in the occurrence and progression of RCC (31–33).

So far, it was reported varying expressions of *ACAT1, BDH2*, and *HMGCL* genes in different types of tumors. Several other enzymes involved in ketogenic pathways were reported to be up-regulated in high-grade prostate cancers and could serve as potential tissue biomarkers (9).

ACAT1 catalyzes the reversible conversion between acetoacetyl-CoA and two molecules of acetyl-CoA. Mutations in *ACAT1* gene lead to 3-ketothiolase deficiency (3KTD) (34). In prostate and breast cancers *ACAT1* expression was reported to be significantly greater compared to adjacent benign tissues (35, 36). Consistent with our finding, White et al. and Zhao et al., using quantitative proteomic analysis, found that ACAT1 protein level was decreased in ccRCC (37, 38). BDH2 acts as a 3-hydroxybutyrate dehydrogenase to convert β-hydroxybutyrate to acetoacetate in liver. This is the first step of ketone body degradation (39). Downregulation of *BDH2* gene expression in liver cancer causes decreased secretion of acetoacetate, leading to inability to prevent recruitment of tumor-associated macrophage, a step in oncogenic transformation (40). On the other hand, in acute myeloid leukemia (AML) patients BDH2 functions as an anti-apoptotic factor, high expression of which serves as an unfavorable prognostic factor in this disease (41). BDH2 was found to be elevated and positively correlated with TNM stages in patients with esophageal cancers as well (42). HMGCL protein belongs to the β-lyase family, is localized to mitochondria in cells and catalyzes the last step in leucine degradation and plays a key role in ketone body production. HMGCL is linked to cancer as well. HMGCL has been reported to be overexpressed in stromal cells of breast cancer. This suggests that ketones produced in the stroma may play a role in tumorigenesis (43). It was found that the proto-oncogene *BRAF V600E* mutant protein was responsible for the up-regulated the *HMGCL* gene expression, leading to increased production of acetoacetate. The increased production of acetoacetate activates MEK-ERK signaling pathway (44), indicating an oncogenic role of ketone bodies. In contrast to this, our previous study showed that *HMGCL* gene is silenced in nasopharyngeal carcinoma (NPC) if compared to normal epithelium. This led us to suggest that HMGCL functions as a potential tumor suppressor in NPC by increasing the intracellular β-hydroxybutyrate (45). Here, we identified that overexpressed *ACAT1, BDH2*, and *HMGCL* genes, remarkably suppresses the proliferative and invasive capacity of ccRCC cells. Except for *HMGCL*, restoration of *ACAT1* and *BDH2* expression impeded the migratory ability of ccRCC cells. Further studies are needed to reveal the underlying molecular mechanisms of the contribution of impaired ketogenesis to ccRCC pathogenesis.

## Conclusions

Our findings from the present study point to a prognostic significance of expression and activity level values for *ACAT1, BDH2*, and *HMGCL* genes, which regulate the ketone body metabolism in ccRCC. The results from our study not only provide new insight into the mechanism of ccRCC progression but also provide useful information which may aid clinicians to design more personalized treatment for patients with ccRCC.

## Data Availability Statement

The datasets used/analyzed during the current study are available from the corresponding author on reasonable request.

## Ethics Statement

The studies involving human participants were reviewed and approved by Ethical permission for this study was granted by the Research Ethics Committee of the Guangxi Medical University (Nanning, China) (document NO. 2019-SB-118). The patients/participants provided their written informed consent to participate in this study.

## Author Contributions

WC and WL performed the experiments. XiaohZ and YLu cultured the cells. WX carried out the meta-analysis. SZ, GF, and YLi performed the bioinformatic analysis. LL, YM, and XX performed the statistical analysis of experimental data. LM was responsible for revising manuscript. GH, ZZ, PL, and XiaoyZ conceived the study and participated in its design. All authors read and approved the final manuscript.

### Conflict of Interest

The authors declare that the research was conducted in the absence of any commercial or financial relationships that could be construed as a potential conflict of interest.

## Abbreviations

RCC: Renal cell carcinoma
ccRCC: Clear cell renal cell carcinoma
CPT-1: Carnitine palmitoyltransferase
ACAT: acetyl-CoA acetyltransferase
HMGCS: HMG-CoA synthase
HMGCL: HMG-CoA lyase
BDH: β-hydroxybutyrate dehydrogenase
DEGs: Differentially expressed genes
GEO: Gene expression omnibus
HP: Horseradish peroxidase
IRS: immunoreactivity score
qRT-PCR: quantitative real-time PCR
Ct: Comparative threshold cycle
IHC: Immunohistochemical
ROC: Receiver operating characteristic curve
AUC: Area under the curve
OS: Overall survival
DFS: Disease-free survival
HIF-1α: Hypoxia-induced factor α
TCA: Tricarboxylic acid
SDH: Succinate dehydrogenase
3KTD: 3-Ketothiolase deficiency
AML: Acute myeloid leukemia
NPC: Nasopharyngeal carcinoma.
