# Supplementary material for: Dysregulation of Ketone Body Metabolism Is Associated With Poor Prognosis for Clear Cell Renal Cell Carcinoma Patients
Source: Front Oncol. 2019 Dec 17;9:1422. doi: 10.3389/fonc.2019.01422 (PMC6928137; doi:10.3389/fonc.2019.01422)
Supplement: Supplementary file 1 [file Table_1.DOCX]

**Supplementary Materials**

**1. Supplementary Materials and Methods**

**A meta-analysis based on GEO database**

Microarray profiles were screened in GEO website using the following search strategy: (cancer* OR carcinoma OR adenocarcinoma OR tumor OR malignant* OR neoplasm* OR oncology*) AND (renal OR kidney). Criteria were set to improve accuracy and normativity: (1) gene expression data were extracted from homo sapiens; (2) samples were all obtained from malignant tissues or uncancerous renal tissues; (3) both healthy and ccRCC groups are comprised of at least three cases; (4) patients involved didn’t receive treatment.

Subsequently, mRNA expression of *ACAT1*, *BDH2,* and *HMGCL* in ccRCC and control groups were retrieved from each GEO chips and log_2_-transformed to calculate the standard mean difference (SMD) and 95% confidential interval (CI) in STATA 12.0 software. Heterogeneity among different researches was evaluated via a chi-squared test of Q and the I^2^ statistic. Evident heterogeneity (P< 0.1 and I^2^> 50%) requires a random-effect model, otherwise (P≤ 0.1 and I^2^≤ 50%), the fixed-effect model should be adopted. To investigate the origin of heterogeneity, a sensitivity analysis was performed in which a single study is excluded at one time. Additionally, Egger’s test was conducted to assess publication bias.

**2. Supplementary Tables**

**Table S1. *ACAT1*, *BDH2* and *HMGCL* expression in clear cell renal cell carcinoma (ccRCC) and non-cancerous studies from the enrolled Gene Expression Omnibus datasets.**

| **Datasets** | **Year** | **Ref** | **ACAT1** | | | | **BDH2** | | | | **HMGCL** | | | |
| --- | --- | --- | --- | --- | --- | --- | --- | --- | --- | --- | --- | --- | --- | --- |
|  |  |  | **ccRCC** | | **Control** | | **ccRCC** | | **Control** | | **ccRCC** | | **Control** | |
|  |  |  | **N** | **Mean ± SD** | **N** | **Mean ± SD** | **N** | **Mean ± SD** | **N** | **Mean ± SD** | **N** | **Mean ± SD** | **N** | **Mean ± SD** |
| GSE6344 | 2006 | [1] | 10 | 11.922±0.629 | 10 | 13.663±0.205 | 10 | 11.092±0.621 | 10 | 12.684±0.304 | 10 | 10.310±0.280 | 10 | 11.485±0.192 |
| GSE11024 | 2008 | [2] | 10 | 11.614±0.299 | 12 | 12.436±0.530 | 10 | 7.526±0.306 | 12 | 8.254±0.524 | 10 | 10.086±0.299 | 12 | 10.609±0.237 |
| GSE11151 | 2008 | [3] | 27 | 12.451±1.051 | 5 | 13.160±1.493 | 27 | 12.384±0.982 | 5 | 13.393±1.009 | 27 | 11.009±0.604 | 5 | 11.361±0.546 |
| GSE12606 | 2008 | [4] | 6 | 7.890±0.613 | 4 | 10.150±0.137 | 6 | 8.008±0.561 | 4 | 10.283±0.230 | 6 | 6.376±0.221 | 4 | 7.462±0.886 |
| GSE14994 | 2009 | [5] | 59 | 11.030±0.763 | 11 | 12.742±0.350 | 59 | 10.883±0.503 | 11 | 12.303±0.336 | 59 | 8.834±0.439 | 11 | 9.912±0.506 |
| GSE15641 | 2009 | [6] | 32 | 9.814±0.918 | 23 | 10.605±0.487 | 32 | 9.350±0.789 | 23 | 9.973±0.521 | 32 | 9.218±0.238 | 23 | 9.007±0.268 |
| GSE16449 | 2010 | [7] | 52 | -0.264±0.885 | 18 | 1.809±0.719 | 52 | 1.611±0.710 | 18 | 2.856±0.802 | 52 | 0.631±0.938 | 18 | 0.278±0.542 |
| GSE17895 | 2010 | [8] | 138 | 10.806±1.137 | 22 | 11.813±0.974 | 138 | 7.281±0.809 | 22 | 8.017±0.630 | 138 | 9.320±0.528 | 22 | 9.873±0.673 |
| GSE26574 | 2011 | [9] | 8 | 11.737±0.465 | 10 | 12.602±0.585 | 8 | 7.741±0.557 | 10 | 7.359±0.502 | 8 | 10.255±0.287 | 10 | 10.609±0.454 |
| GSE36895 | 2012 | [10] | 29 | 11.566±1.087 | 23 | 13.002±0.421 | 29 | 11.520±0.950 | 23 | 13.034±0.417 | 29 | 9.982±0.511 | 23 | 10.934±0.410 |
| GSE40435 | 2013 | [11] | 101 | 11.372±0.790 | 101 | 13.200±0.440 | 101 | 7.061±0.280 | 101 | 7.696±0.292 | 101 | 8.148±0.308 | 101 | 9.225±0.367 |
| GSE47032 | 2013 | [12] | 10 | 7.789±1.143 | 10 | 8.982±0.181 | 10 | 10.155±0.738 | 10 | 11.547±0.301 | 10 | 7.324±0.813 | 10 | 8.816±0.731 |
| GSE53000 | 2014 | [13] | 52 | 9.734±0.790 | 6 | 9.750±0.234 | 52 | 8.016±0.665 | 6 | 8.548±0.291 | 52 | 8.442±0.430 | 6 | 9.040±0.489 |
| GSE53757 | 2014 | [14] | 72 | 12.473±0.772 | 72 | 14.398±0.626 | 72 | 12.571±0.644 | 72 | 14.487±0.552 | 72 | 10.676±0.316 | 72 | 11.705±0.423 |
| GSE76351 | 2015 | NR | 12 | 7.862±0.785 | 12 | 9.891±0.471 | 12 | 5.998±0.677 | 12 | 8.008±0.515 | 12 | 5.998±0.677 | 12 | 8.008±0.515 |
| GSE66270 | 2016 | [15] | 14 | -1.006±0.534 | 14 | 0.498±0.166 | 14 | -0.932±0.525 | 14 | 0.731±0.215 | 14 | -0.567±0.378 | 14 | 0.396±0.204 |
| GSE71963 | 2016 | [16] | 32 | 0.551±0.345 | 16 | 1.902±1.568 | 32 | 0.534±0.323 | 16 | 1.204±0.598 | 32 | 0.627±0.277 | 16 | 1.358±0.751 |
| GSE100666 | 2017 | NR | 3 | 9.467±1.346 | 3 | 12.237±0.951 | 3 | 6.419±1.033 | 3 | 7.675±0.488 | 3 | 10.532±0.405 | 3 | 11.497±0.496 |
| GSE105288 | 2018 | [17] | 35 | 11.513±1.102 | 9 | 12.465±0.702 | 35 | 7.132±0.334 | 9 | 7.477±0.359 | 35 | 8.724±0.505 | 9 | 9.054±0.634 |
| GSE117890 | 2019 | [18] | 5 | 9.834±1.319 | 6 | 10.563±0.473 | 5 | 2.416±1.495 | 6 | -0.909±3.490 | 5 | 8.160±0.558 | 6 | 7.504±0.356 |

Abbreviation: N, number; SD, standard deviation; NR, no reference

**Table S2. The AUC values analysis for mRNA expression of ketone body metabolism-related genes in renal cancer patients.**

| **Gene** | **AUC** | ***p*-value** | **95%CI** | **cut-off value** | **sensitivity** | **specificity** |
| --- | --- | --- | --- | --- | --- | --- |
| ACAT1 | 0.893 | 0.000*** | 0.851-0.935 | 0.715 | 0.882 | 0.833 |
| BDH2 | 0.882 | 0.000*** | 0.842-0.921 | 0.614 | 0.850 | 0.764 |
| HMGCL | 0.847 | 0.000*** | 0.797-0.897 | 0.558 | 0.766 | 0.792 |
| ACAT1 + BDH2 | 0.912 | 0.000*** | 0.874-0.951 | 0.714 | 0.811 | 0.903 |
| ACAT1 + HMGCL | 0.893 | 0.000*** | 0.850-0.937 | 0.673 | 0.867 | 0.806 |
| BDH2 + HMGCL | 0.891 | 0.000*** | 0.850-0.932 | 0.620 | 0.745 | 0.875 |
| ACAT1 + BDH2 + HMGCL | 0.913 | 0.000*** | 0.874-0.952 | 0.856 | 0.838 | 0.847 |

Abbreviation: AUC, area under curve; 95%CI, 95% confidence interval.

*** p<0.001.

**Table S3. Association of ACAT1, BDH2 and HMGCL protein expression with clinicopathological characteristics based on tissue microarray data.**

| **Characteristic** | **ACAT1** | | | **BDH2** | | | **HMGCL** | | |
| --- | --- | --- | --- | --- | --- | --- | --- | --- | --- |
|  | **Negative**  **Cases** | **Positive**  **Cases** | ***p*** | **Negative**  **Cases** | **Positive**  **Cases** | ***p*** | **Negative**  **Cases** | **Positive**  **Cases** | ***p*** |
| Age(years) | | | | | | | | | |
| ＜60 | 31 | 13 | 0.251 | 8 | 35 | 0.745 | 32 | 12 | 0.168 |
| ≥60 | 24 | 17 |  | 9 | 33 |  | 24 | 17 |  |
| Sex | | | | | | | | | |
| Male | 31 | 16 | 0.778 | 11 | 37 | 0.444 | 32 | 15 | 0.634 |
| Female | 24 | 14 |  | 6 | 31 |  | 24 | 14 |  |
| Tumor diameter | | | | | | | | | |
| ≤5cm | 31 | 24 | 0.392 | 3 | 41 | 0.002^**^ | 31 | 13 | 0.357 |
| ＞5cm | 14 | 16 |  | 14 | 27 |  | 25 | 16 |  |
| TNM Stage | | | | | | | | | |
| Ⅰ-Ⅱ | 48 | 23 | 0.208 | 11 | 60 | 0.019^*^ | 52 | 19 | 0.004  ^**^ |
| Ⅲ-Ⅳ | 7 | 7 |  | 6 | 8 |  | 5 | 10 |  |

Negative cases: intensity level of staining equal to 0.

Positive cases: intensity level of staining more than 0

* p<0.05; ** p<0.01; *** p<0.001.

**3. Supplementary Figure legend**

**Figure S1. meta-analysis reveals downregulation of *ACAT1, BDH2,* and *HMGCL* in clear cell renal cell carcinoma(ccRCC) patients compared to healthy cases.** (A-C) Forest plot of *ACAT1*, *BDH2* and *HMGCL* expression in ccRCC over normal kidney controls. (D-F) Sensitivity analysis of meta-analysis for *ACAT1*, *BDH2* and *HMGCL* genes, respectively. (G-I) Egger’s publication test plot of meta-analysis for *ACAT1*, *BDH2* and *HMGCL* genes, respectively.

**4. Supplementary References**

1. Tun HW, Marlow LA, von Roemeling CA et al. Pathway signature and cellular differentiation in clear cell renal cell carcinoma. PLoS One 2010; 5: e10696.

2. Kort EJ, Farber L, Tretiakova M et al. The E2F3-Oncomir-1 axis is activated in Wilms' tumor. Cancer Res 2008; 68: 4034-4038.

3. Yusenko MV, Ruppert T, Kovacs G. Analysis of differentially expressed mitochondrial proteins in chromophobe renal cell carcinomas and renal oncocytomas by 2-D gel electrophoresis. Int J Biol Sci 2010; 6: 213-224.

4. Stickel JS, Weinzierl AO, Hillen N et al. HLA ligand profiles of primary renal cell carcinoma maintained in metastases. Cancer Immunol Immunother 2009; 58: 1407-1417.

5. Beroukhim R, Brunet JP, Di Napoli A et al. Patterns of gene expression and copy-number alterations in von-hippel lindau disease-associated and sporadic clear cell carcinoma of the kidney. Cancer Res 2009; 69: 4674-4681.

6. Jones J, Otu H, Spentzos D et al. Gene signatures of progression and metastasis in renal cell cancer. Clin Cancer Res 2005; 11: 5730-5739.

7. Brannon AR, Reddy A, Seiler M et al. Molecular stratification of clear cell renal cell carcinoma by consensus clustering reveals distinct subtypes and survival patterns. 2010; 1: 152-163.

8. Ding Y, Huang D, Zhang Z et al. Combined gene expression profiling and RNAi screening in clear cell renal cell carcinoma identify PLK1 and other therapeutic kinase targets. Cancer Res 2011; 71: 5225-5234.

9. Ooi A, Wong JC, Petillo D et al. An antioxidant response phenotype shared between hereditary and sporadic type 2 papillary renal cell carcinoma. Cancer Cell 2011; 20: 511-523.

10. Pena-Llopis S, Vega-Rubin-de-Celis S, Liao A et al. BAP1 loss defines a new class of renal cell carcinoma. Nat Genet 2012; 44: 751-759.

11. Wozniak MB, Le Calvez-Kelm F, Abedi-Ardekani B et al. Integrative genome-wide gene expression profiling of clear cell renal cell carcinoma in Czech Republic and in the United States. PLoS One 2013; 8: e57886.

12. Valletti A, Gigante M, Palumbo O et al. Genome-wide analysis of differentially expressed genes and splicing isoforms in clear cell renal cell carcinoma. PLoS One 2013; 8: e78452.

13. Gerlinger M, Horswell S, Larkin J et al. Genomic architecture and evolution of clear cell renal cell carcinomas defined by multiregion sequencing. Nat Genet 2014; 46: 225-233.

14. von Roemeling CA, Radisky DC, Marlow LA et al. Neuronal pentraxin 2 supports clear cell renal cell carcinoma by activating the AMPA-selective glutamate receptor-4. Cancer Res 2014; 74: 4796-4810.

15. Liep J, Kilic E, Meyer HA et al. Cooperative Effect of miR-141-3p and miR-145-5p in the Regulation of Targets in Clear Cell Renal Cell Carcinoma. PLoS One 2016; 11: e0157801.

16. Takahashi M, Tsukamoto Y, Kai T et al. Downregulation of WDR20 due to loss of 14q is involved in the malignant transformation of clear cell renal cell carcinoma. Cancer Sci 2016; 107: 417-423.

17. Nam HY, Chandrashekar DS, Kundu A et al. Integrative Epigenetic and Gene Expression Analysis of Renal Tumor Progression to Metastasis. Mol Cancer Res 2019; 17: 84-96.

18. Lucarelli G, Rutigliano M, Sallustio F et al. Integrated multi-omics characterization reveals a distinctive metabolic signature and the role of NDUFA4L2 in promoting angiogenesis, chemoresistance, and mitochondrial dysfunction in clear cell renal cell carcinoma. Aging (Albany NY) 2018; 10: 3957-3985.
