# Supplementary figures and images for: Dysregulation of Ketone Body Metabolism Is Associated With Poor Prognosis for Clear Cell Renal Cell Carcinoma Patients
Source: Front Oncol. 2019 Dec 17;9:1422. doi: 10.3389/fonc.2019.01422 (PMC6928137; doi:10.3389/fonc.2019.01422)

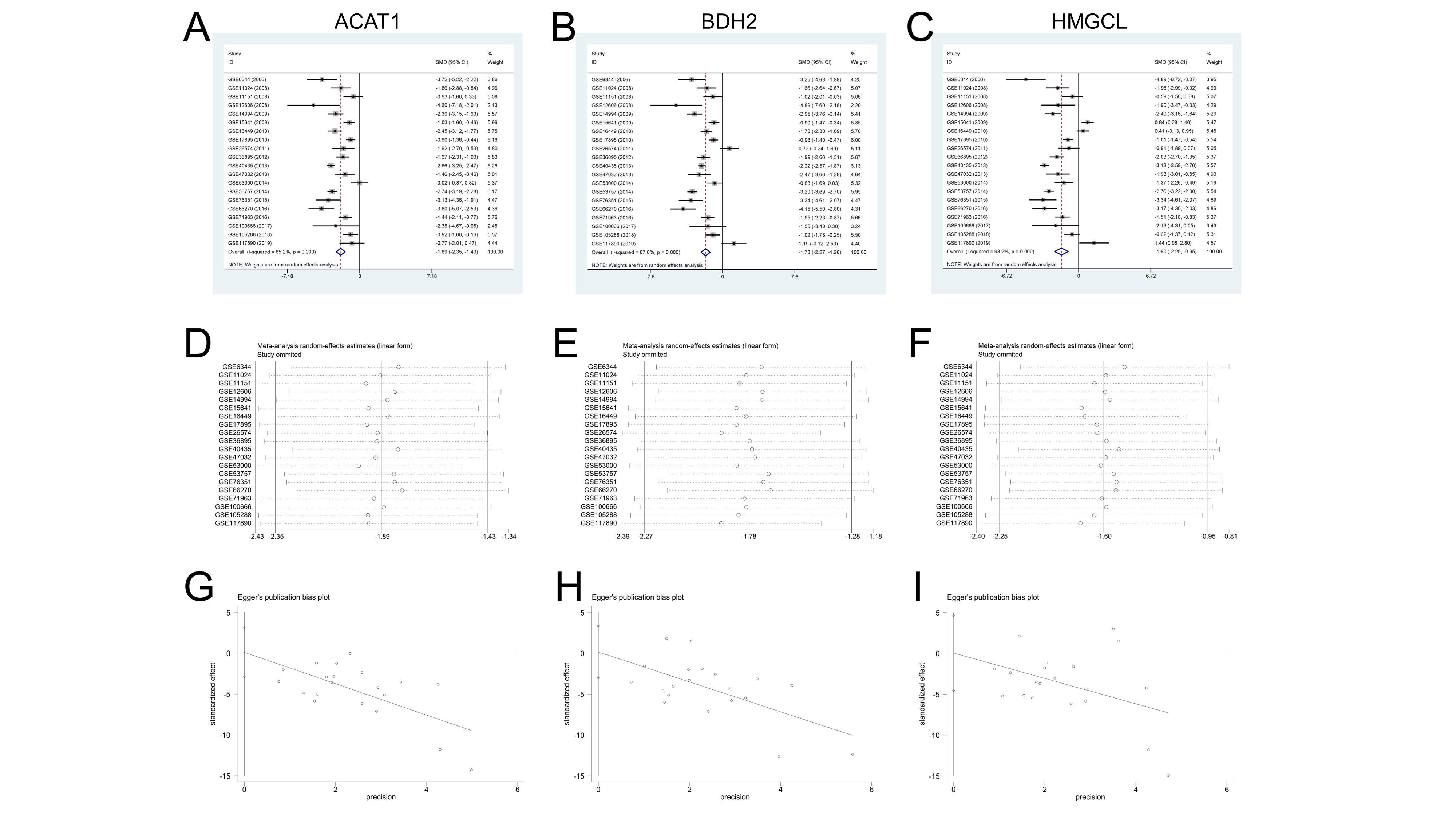

Supplement: Supplementary file 2 [file Image_1.TIF]
